# Adipose knockout of H-ferritin improves energy metabolism in mice

**DOI:** 10.1016/j.molmet.2024.101871

**Published:** 2024-01-05

**Authors:** Binyu Lu, Shanshan Guo, Jialin Zhao, Xiaoting Wang, Bing Zhou

**Affiliations:** 1 Key Laboratory of Quantitative Synthetic Biology, Shenzhen Institute of Synthetic Biology, Shenzhen Institutes of Advanced Technology, Chinese Academy of Sciences, Shenzhen 518055, China; 2 Faculty of Synthetic Biology, Shenzhen Institute of Advanced Technology, Chinese Academy of Sciences, Shenzhen 518055, China

**Keywords:** Fth1, Reactive oxygen species, Iron homeostasis, Adipocyte, Glucose metabolism

## Abstract

**Objective:**

Ferritin, the principal iron storage protein, is essential to iron homeostasis. How iron homeostasis affects the adipose tissue is not well understood. We investigated the role of ferritin heavy chain in adipocytes in energy metabolism.

**Methods:**

We generated adipocyte-specific ferritin heavy chain (*Fth*, also known as *Fth1*) knockout mice, herein referred to as *Fth*^*AKO*^. These mice were analyzed for iron homeostasis, oxidative stress, mitochondrial biogenesis and activity, adaptive thermogenesis, insulin sensitivity, and metabolic measurements. Mouse embryonic fibroblasts and primary mouse adipocytes were used for in vitro experiments.

**Results:**

In *Fth*^*AKO*^ mice, the adipose iron homeostasis was disrupted, accompanied by elevated expression of adipokines, dramatically induced heme oxygenase 1(*Hmox1*) expression, and a notable decrease in the mitochondrial ROS level. Cytosolic ROS elevation in the adipose tissue of *Fth*^*AKO*^ mice was very mild, and we only observed this in the brown adipose tissue (BAT) but not in the white adipose tissue (WAT). *Fth*^*AKO*^ mice presented an altered metabolic profile and showed increased insulin sensitivity, glucose tolerance, and improved adaptive thermogenesis. Interestingly, loss of ferritin resulted in enhanced mitochondrial respiration capacity and a preference for lipid metabolism.

**Conclusions:**

These findings indicate that ferritin in adipocytes is indispensable to intracellular iron homeostasis and regulates systemic lipid and glucose metabolism.

## Highlights

• Fth deletion reduces total iron but increases labile iron, accompanied by induction of some antioxidant genes.
• Adipocyte Fth knockout mice exhibit an altered energy metabolism profile, with a preference for fat metabolism to glucose.
• Adipocyte Fth knockout mice significantly excel in insulin sensitivity and glucose tolerance tests.

## Abbreviations

AT: Adipose tissue
ETC: Electron transport chain
eWAT: Epididymal white adipose tissue
FAO: Fatty acid oxidation
Fpn: Ferroportin
FTH: Ferritin heavy chain
*Fth*^*AKO*^: Adipocyte-specific Fth knockout
*Fth*^*fl/fl*^: Homozygous Fth floxed
FTL: Ferritin light chain
HO-1, Hmox1: Heme oxygenase 1
iBAT: Interscapular brown adipose tissue
iWAT: Inguinal white adipose tissue
LIP: Labile iron pool
mtROS: Mitochondrial reactive oxygen species
ND: Normal diet
RER: Respiratory exchange ratio
ROS: Reactive oxygen species
Tfrc: Transferrin receptor
VE: Vitamin E

## 1 Introduction

Iron is an essential micronutrient involved in various physiological processes. It promotes energy production as an essential component of many enzymes and plays a critical role in cell differentiation and proliferation. Meanwhile, excessive iron can generate reactive oxygen species (ROS) via the Fenton reaction and even lead to ferroptosis, which is the programmed cell death characterized by iron-dependent accumulation of lipid peroxidation. Therefore, iron concentration is strictly regulated in all species. Recent studies have suggested a relationship between iron, energy metabolism [1], and thermogenic functions [[2], [3], [4]]. However, the exact connection has never been clarified. Some data are even incongruent with each other: some work reported that iron increases insulin resistance [5,6], but meanwhile, transferrin receptor gene (*Tfrc*) deletion in mouse adipocytes results in decreased iron in adipocytes, accompanied by insulin insensitivity, increased adiposity, and defective thermogenesis [4]. There seems not to be a simple or direct relationship between them. One important reason for this confusion might be that the already complex connection between iron and energy metabolism is further exacerbated by the fact that both iron and glucose/fat metabolisms are subject to systemic regulation. For example, fat metabolism may be regulated not only by iron in the adipocyte but also by cross-talks with the liver [7], CNS [8], and gut [9,10], where iron all exert effects. A hypothetical scenario to illustrate this is that, on the one hand, iron outside of the adipocytes could act in such a way that directly stimulates fat synthesis [11] or diet intake [12]; however, on the other hand, iron in the adipocyte could influence oppositely, i.e., increase fat utilization [3,13]. The multiple facets of systemic energy regulation by iron thus make conclusions from different studies sometimes hard to reconcile. To simplify matters, focusing on one aspect of iron and energy metabolism in a particular tissue appears necessary.

Ferritin consists of two types of subunits: ferritin heavy chain (FTH) and ferritin light chain (FTL). As the major iron-storage molecule, ferritin serves a vital function in regulating free iron levels and reducing ROS production. Both the increased free iron level and the oxidative stress could induce ferritin expression [14,15]. Clinically, serum ferritin is a well-known marker of body iron levels and inflammation. It is also correlated to several serum adipokines, including insulin [16], adiponectin [17], leptin [12], resistin [18], and fatty acid binding protein 4 (FABP4) [19], while some of the mechanisms are not fully understood. There is a strong correlation between ferritin and energy metabolism.

Fth germline deletion in mice results in embryonic lethality at a very early stage [20]. Inducible systemic *Fth* gene knockout in adult mice also results in a quick death after induction, accompanied by complete loss of adaptive thermogenesis [21]. The broad inactivation of *Fth* and tissue-specific *Fth* targeting, as reported in other studies [[22], [23], [24]], all created altered iron homeostasis. Interestingly, while conditional knockout of *Fth* in several tissues produced some physiological consequences, such as deletion in macrophages resulting in protective effects on obesity and diabetes [22], none of these lead to severe consequences under normal conditions [23,24]. One hypothesis posits that secreted ferritin may have an iron regulating and iron transport function locally in some tissues [[25], [26], [27]], compensating for the loss of ferritin in other tissues. It is also possible that the key tissues for FTH functions are hitherto not revealed, and the several tissues where *Fth* has been removed are not where *Fth* manifests its critical physiological roles.

At the embryonic stage, *Fth* is early and robustly expressed in the heart and brown fat tissue [20]. While *Fth* deletion in the heart mildly affects the mice, the role of FTH in adipose tissues is not reported. To further explore the FTH function and more precisely analyze the iron-fat connection, we report here the generation of adipocyte-specific *Fth1* deletion (*Fth*^*AKO*^) in the mouse. Adipocyte-specific *Fth1* deletion does not lead to death or severe abnormality either. However, a notable increase in glucose tolerance, insulin sensitivity, and a minor increase in adaptive thermogenesis was observed. Concomitantly, an antioxidant-heme oxygenase 1 (*Hmox1*), mitochondrial respiratory ability, and expression of several adipokines were upregulated. This research contributes to understanding the complex interplay between ferritin and energy metabolism and identifying potential therapeutic targets of metabolic diseases.

## 2 Material and methods

### 2.1 Animals

Mice referred to as *Fth*^*AKO*^ mice are conditional adipocyte *Fth1* knockout (*Adipoq-Cre; Fth*^*fl/fl*^) mice, generated by intercrossing *Fth*^*fl/fl*^ mice (Jackson Laboratory, stock #018063) [28] with mice carrying the adiponectin promoter-cre transgene (Cyagen Biosciences, Suzhou, China) [29]. *Fth*^*fl/fl*^ littermate mice were used as controls. Male mice were used in this study. All mice were housed under specific pathogen-free conditions, 20 °C–26 °C, in a 12-h/12-h light/dark cycle. They were given a normal diet (Beijing Keao Xieli Feed Co., Ltd., China) or a vitamin E-supplemented diet (0.5 g of α-tocopherol [Coolaber, Beijing, China] added in 1 kg normal chow) and water *ad libitum*. All animal protocols were approved by the Institutional Animal Care and Use Committee of Shenzhen Institute of Advanced Technology.

### 2.2 Aconitase activity assay

Adipose tissues were dissected and washed with cold PBS. The fresh tissues were homogenized in cold Triton X-100 lysis buffer (20 mmol/l Tris–HCl, pH 8.0, 137 nmol/l NaCl, 1 % Triton X-100, protease inhibitors) and centrifuged at 12,000×*g*, 4 °C to obtain clear tissue lysates. The protein concentration was measured with a BCA kit (Beyotime, Shanghai, China). 60 μg of protein was added to 200 μl of the reaction buffer (50 mmol/l K_2_HPO_4_, 30 mmol/l citric acid, pH 7.4). The increment of absorbance at wavelength 240 nm within 5 min was detected by a microplate reader (Biotek Synergy H1, Santa Clara, CA, USA) as the relative aconitase activity.

### 2.3 Ferrozine iron assay

Detection of total iron based on the colorimetric reaction of ferrous and ferrozine was performed as described previously [30]. Briefly, iron in tissue lysates or serum samples was released by heating and 37 % HCl treatment, reduced to Fe^2+^ by adding ascorbic acid, and reacted with an excess amount of ferrozine. The absorbance of the iron ferrozine complex was measured at 562 nm. Iron concentration was calculated and normalized to protein concentration.

### 2.4 Western blot

Tissue or cell samples were homogenized in ice-cold lysis buffer (Beyotime) containing protease inhibitors (Roche Diagnostics, Basel, Switzerland) and then centrifuged at 4 °C, 12,000×*g* to remove fat layers and debris. Protein concentration was determined by a BCA protein assay kit (Beyotime). The proteins were denatured by boiling at 95 °C in Laemmi loading buffer (Yeasen, Jiangsu, China) for 5 min. The proteins were separated by SDS-PAGE, transferred onto cellulose acetate membranes or PVDF membranes, and immunoblotted for target proteins. Blots were developed with super sensitive ECL substrate (Beyotime) using a chemiluminescent imager (Tanon 5200, Shanghai, China). The antibodies are listed as follows: anti-FTH (1:1000, Abcam, Cambridge, UK, ab65080), anti-HIF1α (1:500, Santa Cruz Biotech, Dallas, TX, USA, sc-13515), anti-β-actin (1:10,000, Abbkine, Wuhan, China, ABL01010), anti-β-tubulin (1:10,000, Abbkine, ABL01030), anti-mouse secondary antibody (1:10,000, Easybio, Beijing, China, BE0102), anti-rabbit secondary antibody (1:10,000, Easybio, BE0101).

### 2.5 Isolation of mature adipocytes

Adipose tissues of mice were dissected, minced, and put into 1.5 ml centrifuge tubes. 1 ml collagenase type II buffer (collagenase 2 mg/ml, Ca^2+^ 10 mmol/l, 2 % BSA) was added to each tube, incubated at 37 °C for 1–2 h, and vortexed every 10 min. After digestion, the brown and white adipocyte suspension was passed through pre-wetted 70 μm and 200 μm sterile cell strainers respectively. The cell suspension was centrifuged at 50×*g* for 5 min at room temperature. The digestion buffer under the floating adipocyte layer was removed with syringes and needles. The adipocytes were rinsed with medium and centrifuged again at 50×*g* for 5 min. The excess medium was removed, and the adipocytes were left in the tubes for follow-up experiments.

### 2.6 Labile iron pool (LIP) measurement

Mature adipocytes were prepared as described above. The number of cells was determined by a cell counter (Nexcelom, Cellometer Mini, Lawrence, MA, USA). According to the cytosolic LIP measurement described previously [31], 10^6^ cells were incubated with Calcein-AM (Aladdin, Shanghai, China). The basal and deferiprone de-quenched fluorescence intensity was measured by a microplate reader using 488 nm excitation and 517 nm emission.

### 2.7 ROS measurement

Mature adipose cells were isolated as described above. After washing with serum-free medium, 10^6^ cells were incubated with DCFH-DA (10 μmol/l, MCE, Monmouth Junction, NJ, USA) at 37 °C for 30 min in the dark for cytosol ROS measurement. After washing the cells with serum-free medium twice, the fluorescence intensity was measured by flow cytometry (BD FACSCelesta, BD Biosciences, Franklin Lakes, NJ, USA). For mitochondrial ROS measurement, a similar procedure was performed with the mitochondrial ROS probe MitoSOX Red (1 μmol/l, Invitrogen, Carlsbad, CA, USA). The fluorescence intensity was measured with a microplate reader using 396 nm excitation and 610 nm emission.

### 2.8 Mitochondrial ETC complex I and complex II activity assays

Mitochondrial proteins were prepared according to the instructions of the mitochondrial complex I and II activity detection kits (Cominbio, Suzhou, China). The protein concentration of the samples was detected by a BCA kit (Beyotime). The activity of complex I was determined by the oxidation rate of NADH measured by absorbance at 340 nm. The activity of complex II was determined by the reaction rate of 2,6-dichloro-phenol indophenol measured by absorbance at 605 nm. The absorbance was detected by a microplate reader (Biotek, SynergyH1, Agilent). Western blot was performed to detect the expression of complex I and II in samples to verify the purity of the extracted mitochondria.

### 2.9 IPGTT and IPITT

8–10 weeks old mice were tested for glucose tolerance tests (GTTs), and the same mice were tested for insulin tolerance tests (ITTs) 2 weeks later. For GTTs, after fasting for 16 h, each mouse received an i.p. injection of glucose solution (2 g/kg body weight, 20 % wt/vol. glucose in 0.154 mol/l NaCl solution in water), and the blood glucose levels were monitored with a glucometer (Yuwell Accusure 580, Shanghai, China) at 0, 15, 30, 60, 90, and 120 min after injection, respectively. For ITTs, after fasting for 6 h, mice were given i.p. injections of 0.75 IU/kg body weight of insulin (Humalog, VL7516, Elilly, USA; 0.075 IU/ml insulin in 0.154 mol/l NaCl solution in water). The blood glucose levels were measured at the same time points as in GTTs.

### 2.10 Quantitative RT-PCR (qRT-PCR)

RNA was extracted from adipose tissues of mice using TRIzol reagent, then reverse-transcribed to cDNA using a cDNA Synthesis Kit (TRANSGEN, Beijing, China). qRT-PCR was performed using a qRT-PCR reaction kit (TRANSGEN) on a Bio-rad CFX connect detection system (Biorad, Hercules, CA, USA), using 4–6 independent RNA samples as biological replicates. The mRNA expression of the target genes was normalized to the expression of *18S rRNA* or *Arbp0*, and the values were compared with the control group. The primer sequences are shown in Suppl. Table S1.

### 2.11 Malondialdehyde (MDA) assay

Adipose tissues were homogenized in PBS and centrifuged at 10,000×*g*, 4 °C for 10 min. The fat cakes and the bottom debris were removed. The protein concentration of the samples determined by a BCA kit (Beyotime) was used for subsequent calculation. Malondialdehyde concentration was measured with an MDA assay kit (Beyotime) based on the colorimetric reaction of MDA and thiobarbituric acid producing a red product.

### 2.12 Paraffin embedding, sectioning, Prussian blue and DAB staining, and H&E staining

The dissected tissues were fixed with 4 % paraformaldehyde for 24 h at room temperature. After dehydration, they were paraffin-embedded and sectioned at 8 μm. Paraffin sections on slides were deparaffinized and rehydrated. Tissue sections were flooded with the Prussian blue staining buffer (0.15 g potassium hexacyanoferrate, 0.4 ml 37 % HCl, 7.1 ml H_2_O) for 60 min in the dark, then were washed in H_2_O four changes for 5 min each. Sections were then stained with DAB (diaminobenzidine) in the dark for about 40 min and washed in H_2_O four changes for 5 min each. They were stained shortly with hematoxylin solution and washed well in H_2_O. The slides were dehydrated in graded alcohols and cleared in xylene. They were air-dried and mounted with neutral balsam mounting medium (BBI Life Science, Shanghai, China) and coverslips. The H&E staining procedure was the same as above, except that the sections were shortly stained with hematoxylin solution and eosin solution. The slides were scanned (3DHISTECH, Budapest, Hungary) and analyzed.

### 2.13 Rectal temperature measurement

The tested mouse was held in one hand, and the Vaseline-lubricated probe of a digital rectal thermometer (TH212, Zhongjiao, Cangzhou, China) was gently and fully inserted into the rectum. The temperature was monitored for about 1 min until a steady reading was recorded.

### 2.14 Cold exposure test

Just before sacrifice, 16-17 weeks-old mice were housed one per cage in a 4 °C cold chamber for 24 h (9 a.m.–9 a.m.) with unlimited access to food and water. Rectal temperature was measured before and by the end of cold exposure sessions at the same time of the day.

### 2.15 Serum leptin

Serum leptin concentration was measured by a mouse leptin ELISA kit (ProteinTech, Wuhan, China) according to the manufacturer's instructions.

### 2.16 Metabolic cage studies

8-9 weeks-old mice were housed individually in metabolic cages (Oxymax-CLAMS, Columbus Instruments, OH, USA). The volumes of oxygen consumption (VO_2_) and carbon dioxide production (VCO_2_) of each mouse were recorded for 24 h when they settled down. Respiratory exchange ratio (RER) = VCO_2_/VO_2_.

### 2.17 Statistical analysis

qRT-PCR results were presented as the mean +SD. Other results were presented as the mean ± SD. Images were analyzed using ImageJ. Data analysis was performed using the GraphPad Prism 9 program. Statistical significance was determined by a two-tailed unpaired t-test for comparisons between two groups or by two-way ANOVA followed by Sidak's multiple comparisons unless specifically noted. Differences with a *p*-value <0.05 were considered statistically significant. Representation of the *p*-value was ∗*p* < 0.05, ∗∗*p* < 0.01, ∗∗∗*p* < 0.001, ∗∗∗∗*p* < 0.0001, and ns: not significant (*p* > 0.05).

## 3 Results

### 3.1 Targeted mutagenesis of *Fth1* in the adipose tissue had little effect on the mice's appearance

**Figure 1:**
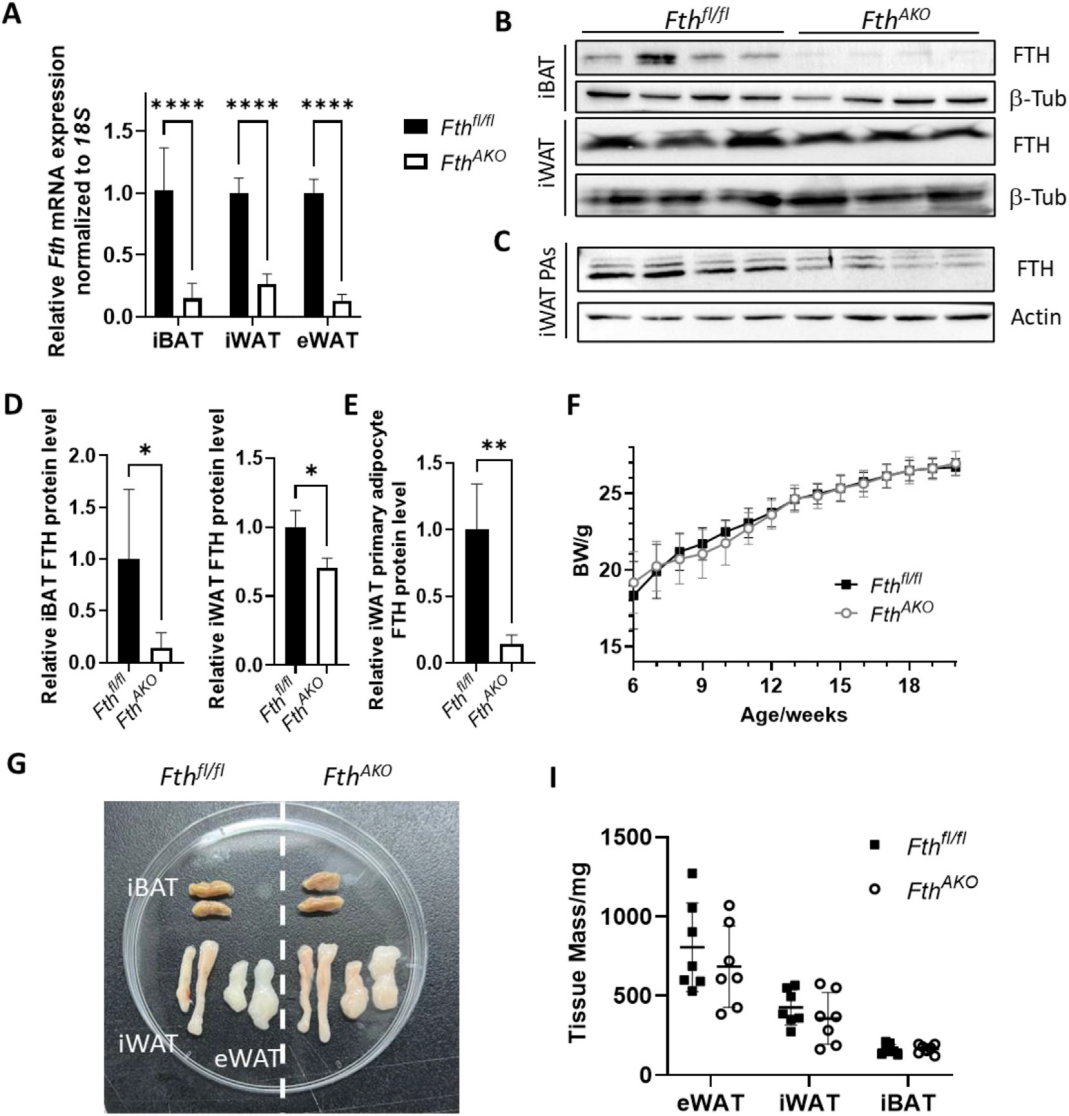
***Fth***^***AKO***^**mice presented no apparent alteration in body weight or fat size.** (A) qRT-PCR analysis of *Fth* expression in iBAT, iWAT, and eWAT of *Fth*^*AKO*^ and *Fth*^*fl/fl*^ mice (*n* = 4, data pooled from two independent experiments). (B,D) Representative western blot images and quantifications of iBAT and iWAT from *Fth*^*AKO*^ and *Fth*^*fl/fl*^ mice (*n* = 3–4, data pooled from two independent experiments). (C,E) Representative western blot images and quantifications of primary adipocytes derived from iWAT (*n* = 4, data pooled from two independent experiments). (F) Body weights of *Fth*^*AKO*^ and *Fth*^*fl/fl*^ mice were measured each week for 14 weeks (*n* = 5). (G) *Fth*^*AKO*^ and *Fth*^*fl/fl*^ mice were sacrificed at 20 weeks, and representative images of iBAT, iWAT, and eWAT were shown. (I) Weights of iBAT, iWAT, and eWAT dissected from *Fth*^*AKO*^ and *Fth*^*fl/fl*^ mice were measured (*n* = 7, from two independent experiments). Data was analyzed by two-tailed unpaired t-tests. ∗*p* < 0.05, ∗∗*p* < 0.01, and ∗∗∗∗*p* < 0.0001.

To investigate the function of ferritin in the adipose tissue (AT), we generated mice with adipocyte-specific deletion of *Fth1* (*Fth*^*AKO*^) by crossing *Fth*^*fl/fl*^ mice [28] with adipoq-cre mice [29]. We found that *Fth1* was successfully deleted in most AT by examining the *Fth1* mRNA expression (Figure 1A). Consistent with the *Fth1* mRNA results, we found that FTH protein expression was greatly reduced in the interscapular brown adipose tissue (iBAT) of *Fth*^*AKO*^ mice as compared to that of control littermates (*Fth*^*fl/fl*^) (Figure 1B,D). However, the FTH protein level in the inguinal white adipose tissue (iWAT) of *Fth*^*AKO*^ mice was only moderately lower than the control. To be certain whether this was due to mixing in some other cell types in the iWAT, we generated primary adipocyte cultures from iWAT of *Fth*^*AKO*^ mice and analyzed their *Fth1* expression. The FTH protein level was dramatically reduced in the cultured primary adipocytes from iWAT of *Fth*^*AKO*^ mice (Figure 1C,E). Therefore, *Fth1* was efficiently removed in both iBAT and iWAT. It might be that *Fth* expression was relatively low in the white fat, making the observed *Fth1* expression more vulnerable to interference from the other cell types. There was no difference between *Fth*^*AKO*^ and the control in the body weight or AT sizes, including those of iBAT, iWAT, and epididymal adipose tissue (eWAT) (Figure 1F,I). Nevertheless, *Fth*^*AKO*^ mice demonstrated a decrease in the adipocyte cell size of eWAT, which was not found in the iBAT or iWAT (Suppl.Fig.S1).

### 3.2 Iron homeostasis of adipose tissue was altered in *Fth*^*AKO*^ mice

**Figure 2:**
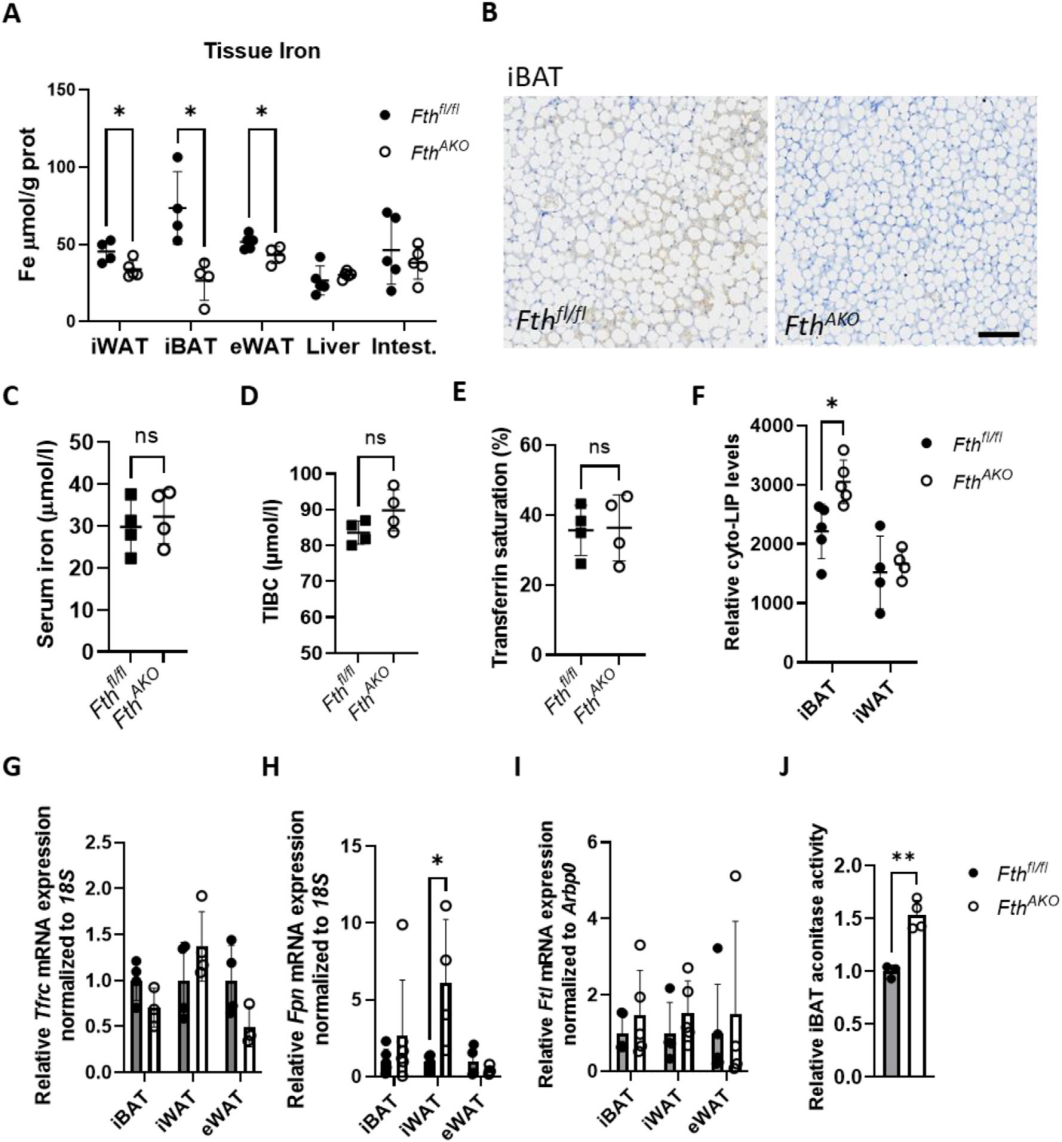
**Iron homeostasis of the adipose tissue was altered in *Fth***^***AKO***^**mice.** (A) Total non-heme iron of the iWAT, iBAT, eWAT, liver, and small intestine in *Fth*^*AKO*^ and *Fth*^*fl/fl*^ mice (*n* = 4–5, from two independent experiments). (B) Representative Perls/DAB iron staining (brown) and hematoxylin staining (blue) images of iBAT of *Fth*^*AKO*^ and *Fth*^*fl/fl*^ mice; scale bar, 100 μm. (C–E) Serum iron, total iron-binding capacity, and transferrin saturation levels of *Fth*^*AKO*^ and *Fth*^*fl/fl*^ mice (*n* = 4). (F) Cytosolic LIP levels of mature adipocytes isolated from iBAT and iWAT were detected with the DCFH-DA probe and normalized by protein concentrations (n = 4–5, from two independent experiments). (G–I) qRT-PCR analysis of *Tfrc*, *Fpn*, and *Ftl* expression in iBAT, iWAT, and eWAT of *Fth*^*AKO*^ and *Fth*^*fl/fl*^ mice (G: *n* = 3–4; H: *n* = 4–6; I: *n* = 4–5, from two independent experiments). (J) Aconitase activity of iBAT of *Fth*^*AKO*^ and *Fth*^*fl/fl*^ mice (*n* = 3–4, from two independent experiments). Data was analyzed by two-tailed unpaired t-tests. ∗*p* < 0.05, ∗∗*p* < 0.01, and ns: not significant (*p* > 0.05). (For interpretation of the references to color/colour in this figure legend, the reader is referred to the Web version of this article.)

Ferritin is a critical intracellular iron storage protein. To examine possible adipose iron disrupted homeostasis in the *Fth*^*AKO*^ mice, we first inspected the total iron level in the fat tissues. We observed that total tissue iron levels were significantly lower in iBAT and slightly lower in iWAT and eWAT, which was almost certainly due to a substantial reduction in the *Fth1* expression (Figure 2A). Perls/DAB iron staining of iBAT also illustrated the same result (Figure 2B). In contrast, no significant change in total iron was observed in the liver, small intestine, or blood serum (Figure 2A,C). Transferrin saturation and total iron binding capacity were unaffected either (Figure 2D,E), showing that *Fth* knockout in the AT does not obviously affect system iron homeostasis. In WATs, total iron levels appeared less affected, likely due to the white fat's intrinsically low iron-storage property. The labile iron pool (LIP) of adipocytes isolated from the iBAT of *Fth*^*AKO*^ mice was higher than that of controls (Figure 2F). However, we failed to detect significant expression change of transferrin receptor (*Tfrc*) and ferritin light chain (*Ftl*) (Figure 2G,J) at the mRNA level. In the iWAT of *Fth*^*AKO*^ mice, the expression of ferroportin (*Fpn*) was higher than that of the control (Figure 2H), implying disrupted labile iron homeostasis. Aconitase activities depend on Fe–S cluster formation, and iron insufficiency reduces the enzymatic activity. We found that the aconitase activity was much higher in the iBAT of *Fth*^*AKO*^ (Figure 2J, Suppl.Fig.S2B), indicating an elevated Fe–S cluster assembly, consistent with the previously observed increased LIP. Collectively, these results suggest that *Fth1* knockout leads to increased available iron in iBAT and iWAT.

### 3.3 Mild oxidative stress without obvious oxidative damage was observed in the AT of *Fth*^*AKO*^ mice

**Figure 3:**
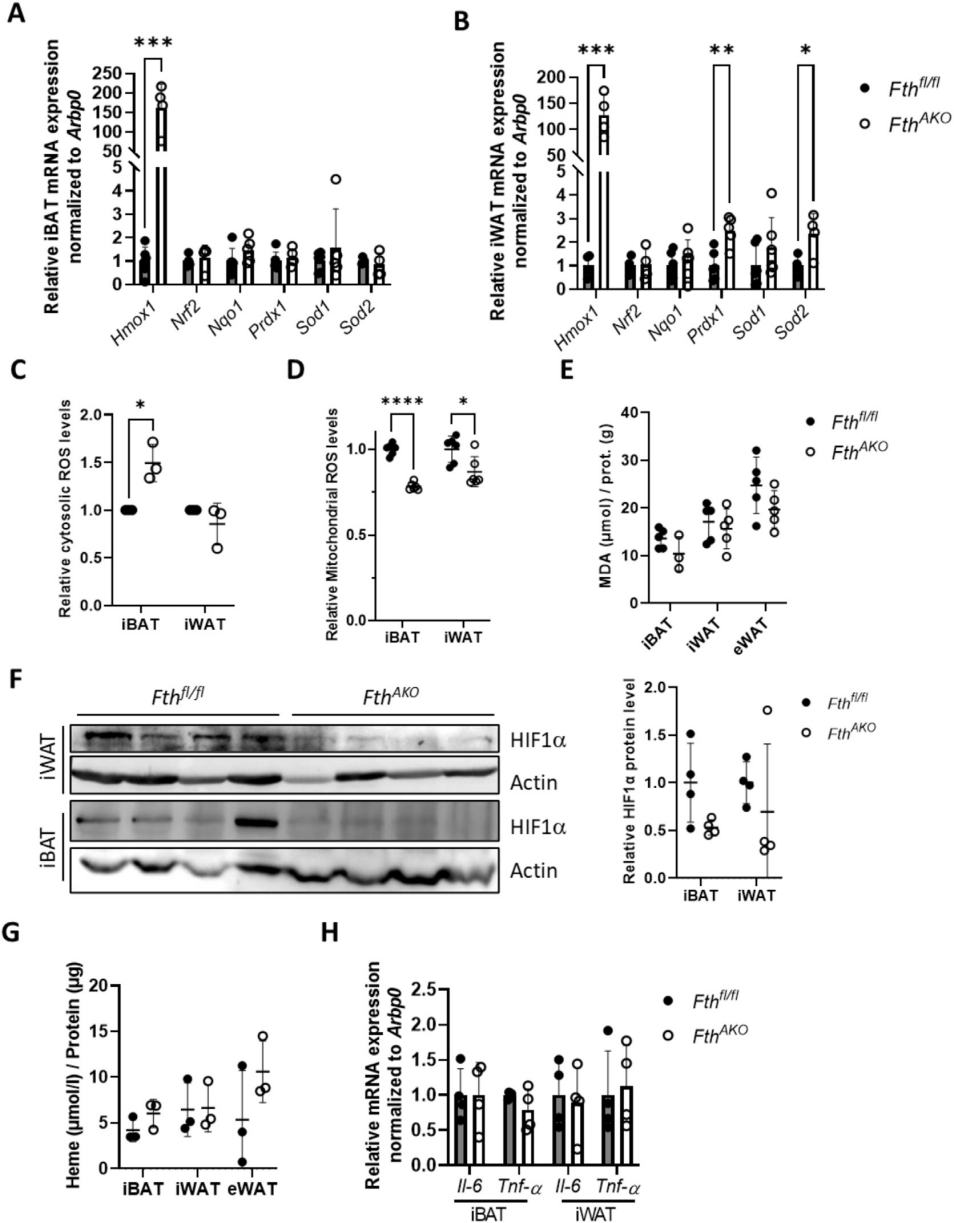
**Fth KO in the adipose tissue induced a mild cytosolic ROS elevation and mitochondrial ROS reduction but no overt lipid oxidation or inflammation.** (A,B) qRT-PCR analysis of *Hmox1*, *Nrf2*, *Nqo1*, *Prdx1*, *Sod1*, and *Sod2* expression in iBAT and iWAT of *Fth*^*AKO*^ and *Fth*^*fl/fl*^ mice (*n* = 4–6, from three independent experiments). (C) Summary data of flow cytometry analysis of cytosolic ROS tested by DCFH-DA probe in iBAT and iWAT adipocytes from *Fth*^*AKO*^ and *Fth*^*fl/fl*^ mice (*n* = 3, from three independent experiments). (D) Mitochondrial ROS level probed by MitoSOX in adipocytes isolated from the iBAT and iWAT of *Fth*^*AKO*^ and *Fth*^*fl/fl*^ mice, normalized to protein content (*n* = 3 mice, from two independent experiments). (E) MDA levels in iBAT, iWAT, and eWAT of *Fth*^*AKO*^ and *Fth*^*fl/fl*^ mice, normalized to protein content (*n* = 3–5, from two independent experiments). (F) Western blots of HIF1α expression in iBAT and iWAT from *Fth*^*AKO*^ and *Fth*^*fl/fl*^ mice. (*n* = 4, from two independent experiments). (G) Heme concentration in iBAT and iWAT of *Fth*^*AKO*^ and *Fth*^*fl/fl*^ mice, normalized to protein content (*n* = 3, from two independent experiments). (H) qRT-PCR analysis of *Il-6* and *Tnf-α* expression in iBAT and iWAT of *Fth*^*AKO*^ and *Fth*^*fl/fl*^ mice (*n* = 4, from two independent experiments). Data from Figure 3C was analyzed by paired t-tests. Other data was analyzed by two-tailed unpaired t-tests. ∗*p* < 0.05, ∗∗*p* < 0.01, ∗∗∗*p* < 0.001, and ∗∗∗∗*p* < 0.0001.

Iron is the primary cellular ROS catalyzer via the Fenton's reaction. As LIP was upregulated in the AT, we next investigated whether ROS production was induced in iBAT and iWAT of *Fth*^*AKO*^ mice. The expression of many oxidative stress-responding genes, including *Nrf2* and its downstream targets, were not affected in iBAT, while peroxiredoxin-1 (*Prdx1*) and superoxide dismutase 2 (*Sod2*) were significantly increased in iWAT (Figure 3A,B). Intriguingly, *Fth1* deletion dramatically elevated the expression of stress-responsive gene heme oxygenase-1 (*Hmox1*) in iBAT, iWAT, and eWAT (Figure 3A,B, Suppl.Fig.S2A). We thus directly measured the cytosolic ROS level in mature adipocytes isolated from iBAT and iWAT. A slight increase in ROS was only observed in the iBAT of *Fth*^*AKO*^ mice (Figure 3C). This slight ROS elevation appeared to produce no significant change in the level of malondialdehyde (MDA), which is the product of lipid peroxidation (Figure 3E). The remarkable increment of *Hmox1* prompted us to examine other factors that could induce its expression. Surprisingly, neither the heme level nor the expression of hypoxia-inducible factor-1α (*Hif1α*) had significantly increased in Fth-deficient ATs compared to the control (Figure 3F,G). We speculated that *Fth1* deletion altered the LIP and caused only very mild oxidative stress, which was insufficient to bring about a broad and robust ROS response. Nevertheless, this low level of ROS was sufficient to stimulate the expression of the antioxidant genes *Prdx1* and *Sod2* in iWAT and *Hmox1* in both iBAT and iWAT. We noticed that *Hmox1* induction in our experiments was about 100-fold, whereas, in *Fth1* global deletion mice, which was associated with strong ROS induction, *Hmox1* induction reached more than 1000-fold [21]. Considering that oxidative stress could cause inflammation, we also examined the expression of inflammation markers and found no changes in the expression of *Il-6* or *Tnf-*α in ATs (Figure 3H).

We, in addition, monitored the mitochondrial ROS (mtROS) level. Interestingly, unlike cytosolic ROS, mtROS slightly declined in adipocytes from iBAT and iWAT of *Fth*^*AKO*^ mice (Figure 3D). In conclusion, our results suggest that *Fth* ablation induced a slight cytosolic ROS elevation but mtROS reduction in the fat. No overt lipid oxidation or inflammation was observed, likely due to the mild nature of the oxidative stress and concomitant stimulated expression of several cytoprotective genes.

### *3.4* *Fth* deletion augmented mitochondrial biogenesis and respirational activity in iWAT, and enhanced body adaptive thermogenesis

**Figure 4:**
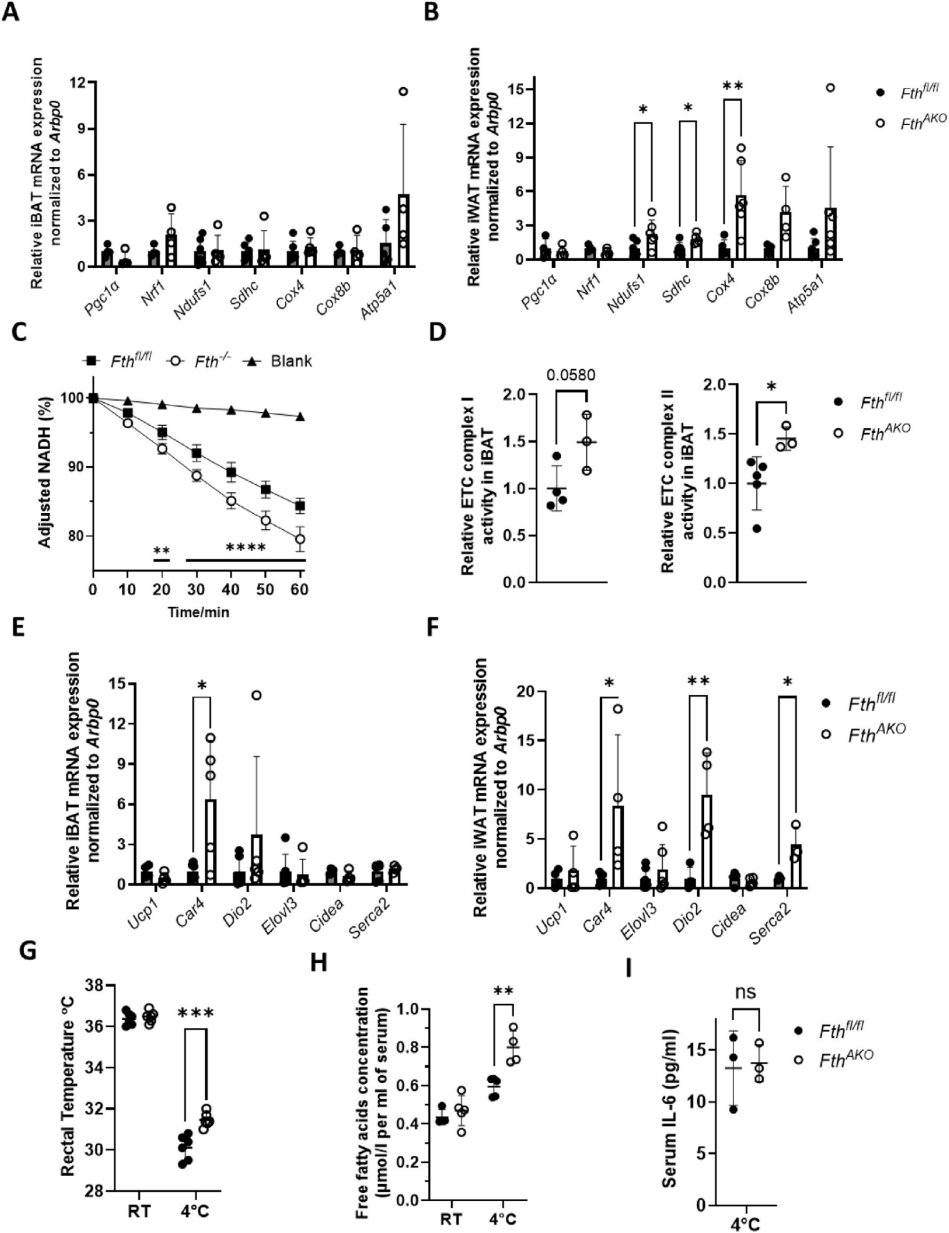
***Fth1* deletion in the adipose tissue caused upregulation of mitochondrial biogenesis and respiratory capacity and a tendency of iWAT browning.** (A,B) qRT-PCR analysis of *Pgc1α, Nrf1, Ndufs1, Sdhc, Cox4, Cox8b,* and *Atp5a1* expression in iBAT and iWAT of *Fth*^*AKO*^ and *Fth*^*fl/fl*^ mice (*n* = 4–6, from two independent experiments). (C) NADH consumption assay of mitochondria isolated from *Fth*^*−/−*^ and *Fth*^*fl/fl*^ MEFs (*n* = 5, from 3 independent experiments). (D) The activities of mitochondrial ETC complex I and II of mitochondria isolated from mature adipocytes of iBAT and iWAT (*n* = 3–5, from two independent experiments). (E,F) qRT-PCR analysis of *Ucp1, Car4, Dio2, Elovl3, Cidea*, and *Serca2* expression in iBAT and iWAT of *Fth*^*AKO*^ and *Fth*^*fl/fl*^ mice (E: *n* = 4–6; F: *n* = 3–6, from three independent experiments). (G) Rectal temperature of *Fth*^*AKO*^ and *Fth*^*fl/fl*^ mice housed at room temperature or 4 °C (*n* = 6, from three independent experiments). (H) Free fatty acid in mice serum before and after cold exposure test (n = 4–5, from two independent experiments). (I) Serum Il-6 of *Fth*^*AKO*^ and *Fth*^*fl/fl*^ mice housed at 4 °C (*n* = 3). Statistical significance of the difference between *Fth*^*−/−*^ and *Fth*^*fl/fl*^ in Figure 4C was determined by two-way ANOVA followed by Sidak's multiple comparisons. Other data was analyzed by two-tailed unpaired t-tests. ∗*p* < 0.05, ∗∗*p* < 0.01, ∗∗∗*p* < 0.001, ∗∗∗∗*p* < 0.0001, and ns: not significant (*p* > 0.05).

It has been proven that *Hmox1* gene expression promotes mitochondrial biogenesis and supports electron transport chain (ETC) activity via activation of *Pgc1, Nrf1*, and *Nrf2* regulated pathways [[32], [33], [34]]. We thus measured the expression levels of *Pgc1α*, *Nrf1, Nrf2*, and ETC genes in ATs. The expression of *Pgc1α*, *Nrf1*, or *Nrf2* was not upregulated. However, the expression of ETC genes (*Cox4* and *Atp5a1*) was significantly enhanced in the Fth-deficient iWAT (Figure 4A,B). To confirm the effect of *Fth* deletion on mitochondrial function, we checked the respiratory activity of mitochondria isolated from *Fth*-deleted mouse embryo fibroblasts (MEFs) by measuring the consumption of respiration substrate NADH. Mitochondria from *Fth*-deleted MEFs exhibited an increased NADH consumption rate compared to those from the control MEFs (Figure 4C). We further tested the activities of mitochondrial ETC complex I and II of mitochondria isolated from iBAT or iWAT adipocytes. In the iBAT of *Fth*^*AKO*^, complex II activity was significantly more robust, while complex I activity also trended higher, though not statistically significant (Figure 4D). We did not observe any difference in activity of complex I or II in the iWAT.

The mitochondrial function and content in ATs are parallel with the browning of white fat and its thermogenesis function. Therefore, we inspected the expression of thermogenic genes and white fat browning markers in iBAT and iWAT. Induced mRNA expression of white fat browning markers *Car4* and *Dio2* was observed in iWAT of *Fth*^*AKO*^ mice, but no significant alteration was seen in iBAT (Figure 4E,F). We then asked whether *Fth* knockout in the ATs affects thermogenesis in mice. We challenged *Fth*^*AKO*^ and control mice with cold stress to elucidate their adaptive thermogenic functions. *Fth*^*AKO*^ mice maintained slightly higher body core temperatures compared to the control in the cold exposure test (Figure 4G). The cold-induced elevation of free fatty acids in the serum was greater in *Fth*^*AKO*^ mice (Figure 4H), suggesting augmented lipolysis in ATs and more fuel consumption for heat production. We measured the IL-6 level in serum to determine if the inflammatory effect contributed to the higher body temperature in cold conditions. The result showed no difference in serum IL-6 level (Figure 4I).

Altogether, the increased mitochondrial biogenesis, mitochondrial function, and lipolysis process in ATs supported *Fth*^*AKO*^ mice to maintain a higher body core temperature in cold environments.

### *3.5* *Fth*^*AKO*^ mice exhibited an altered energy substrate preference, accompanied by increased blood glucose tolerance and insulin sensitivity

**Figure 5:**
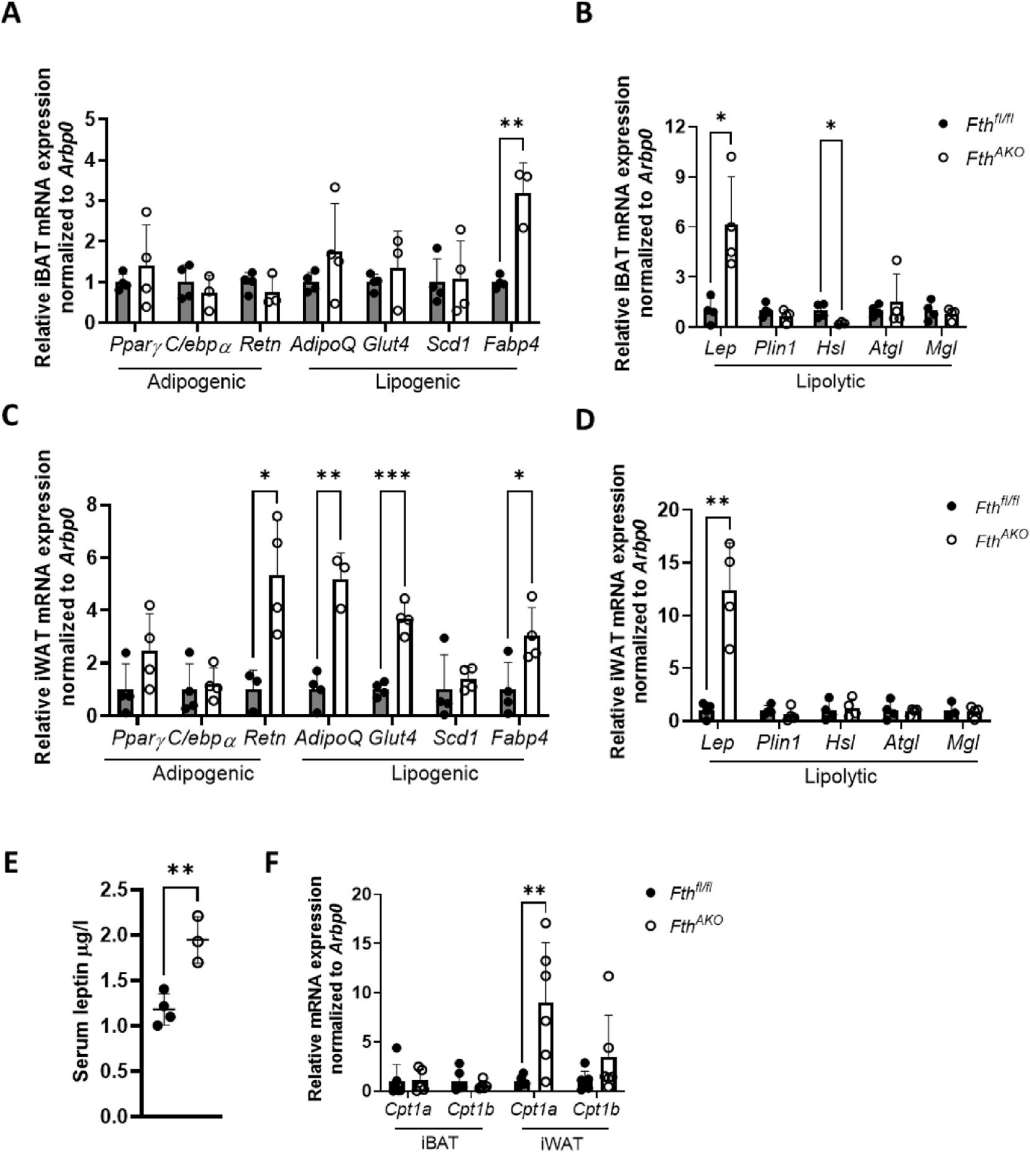
***Fth***^***AKO***^**mice exhibited an altered adipose gene expression profile.** (A,B) qRT-PCR analysis of *Pparγ, C/ebpα, Retn, Plin1, AdipoQ, Glut4, Scd1, Lep, Fabp4, Hsl, Atgl,* and *Mgl* expression in iBAT and iWAT of *Fth*^*AKO*^ and *Fth*^*fl/fl*^ mice (*n* = 3–4, from two independent experiments). (C) Serum leptin of *Fth*^*AKO*^ and *Fth*^*fl/fl*^ mice (*n* = 3–4). (D) qRT-PCR analysis of *Cpt1a* and *Cpt1b* expression in iBAT and iWAT of *Fth*^*AKO*^ and *Fth*^*fl/fl*^ mice (*n* = 5–6, from two independent experiments). Data was analyzed by two-tailed unpaired t-tests. ∗*p* < 0.05, ∗∗*p* < 0.01, and ∗∗∗*p* < 0.001.

An altered metabolic profile under cold stress prompted us to explore more fully possible changes in lipid metabolism with *Fth*^*AKO*^ mice under normal conditions. We therefore used qRT-PCR to analyze expression patterns of some representative adipogenic, lipogenic, and lipogenesis genes. We found that *Fth* deletion significantly upregulated the expression of fatty acid binding protein 4 gene (*Fabp4*) and leptin (*Lep)* and downregulated the expression of hormone-sensitive lipase (*Hsl*) in iBAT (Figure 5A,B). Meanwhile, resistin (*Retn*), adiponectin (*AdipoQ*), glucose transporter type 4 (*Glut4*), *Fabp4*, and *Lep* were significantly upregulated in Fth-deficient iWAT (Figure 5C,D). These results suggest that *Fth* deletion in ATs might lead to elevated fatty acid content in iBAT and iWAT [35] and activated lipogenesis in iWAT. In agreement with gene expression data, we found that serum leptin level was also notably enhanced in *Fth*^*AKO*^ mice (Figure 5E). As leptin and FABP4 have opposite effects on mitochondrial fatty-acid β-oxidation (FAO) function [36], we also tested whether the FAO capacity was enhanced in *Fth*^*AKO*^ mice. The expression of β-oxidation enzyme gene *Cpt1a* was elevated in the iWAT of *Fth*^*AKO*^ mice (Figure 5F).

**Figure 6:**
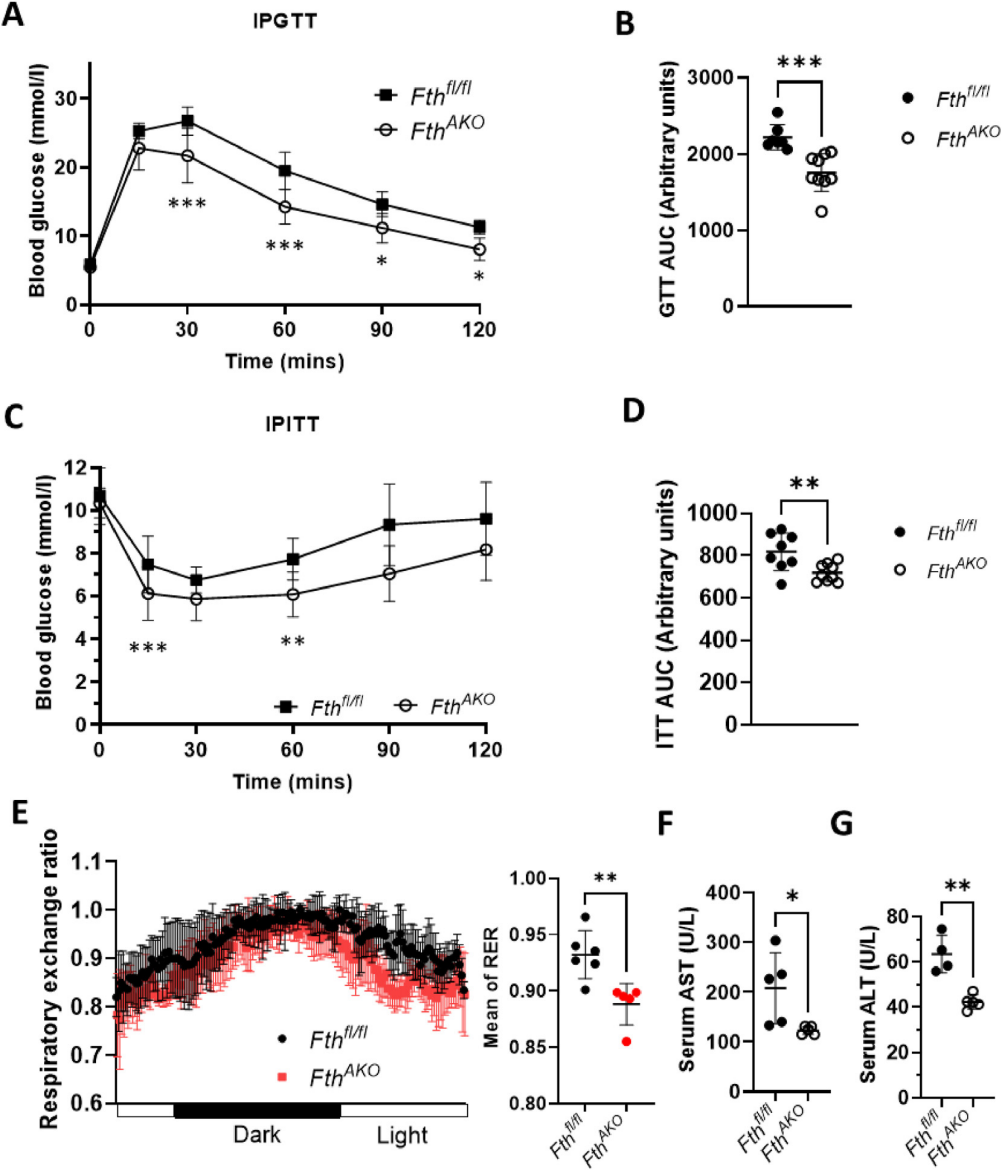
***Fth***^***AKO***^**mice had increased glucose tolerance, insulin sensitivity, and altered energy substrate preference.** (A,B) IPGTT and (C,D) IPITT results of *Fth*^*AKO*^ and *Fth*^*fl/fl*^ mice and AUC calculated from them (*n* = 7–9, from three independent experiments). (E) The respiratory exchange ratio (RER, VCO_2_/VO_2_) of *Fth*^*AKO*^ and *Fth*^*fl/fl*^ mice calculated from VCO_2_ and VO_2_ monitored with metabolism cages over a 24-h period (*n* = 5–6). (F,G) Serum AST and serum ALT concentrations in *Fth*^*AKO*^ and *Fth*^*fl/fl*^ mice (*n* = 4–5, from two independent experiments.). Data from Figure 6A,C was analyzed by two-way ANOVA followed by Sidak's multiple comparisons. Other data was analyzed by two-tailed unpaired t-tests. ∗*p* < 0.05, ∗∗*p* < 0.01, and ∗∗∗*p* < 0.001.

Fat and glucose metabolisms are often interconnected. We next investigated the effect of adipocyte *Fth* deletion on glucose metabolism in mice. We subjected these mice to IPGTTs and IPITTs. *Fth*^*AKO*^ mice exhibited significantly increased glucose tolerance and insulin sensitivity (Figure 6A–D). Furthermore, we found that *Fth*^*AKO*^ mice had a lower average respiratory exchange ratio (RER) compared to control littermates, suggesting a shift of metabolic substrate from carbohydrates toward fats (Figure 6E). Additionally, we tested liver function markers serum AST and ALT, which were also related to insulin resistance [37]. Both serum AST and ALT levels of *Fth*^*AKO*^ mice were significantly lower than the control, though all were within the normal range (Figure 6F,G).

Elevated *Lep, AdipoQ,* and *Glut4* expression levels in *Fth*^*AKO*^ mice were consistent with their increased insulin sensitivity and glucose tolerance [[38], [39], [40]]. Increased expression of *Lep, AdipoQ,* and altered metabolism substrate were in line with the enhanced FAO in Fth-deficient ATs [41,42]. Taken together, our results indicated that *Fth* adipocyte knockout increased the expression of several lipogenic genes in ATs, which was plausibly associated with increased glucose tolerance, insulin sensitivity, and altered energy substrate.

### 3.6 Vitamin E supplementation improved the glucose tolerance and insulin resistance of both *Fth*^*AKO*^ and control mice and diminished their difference

**Figure 7:**
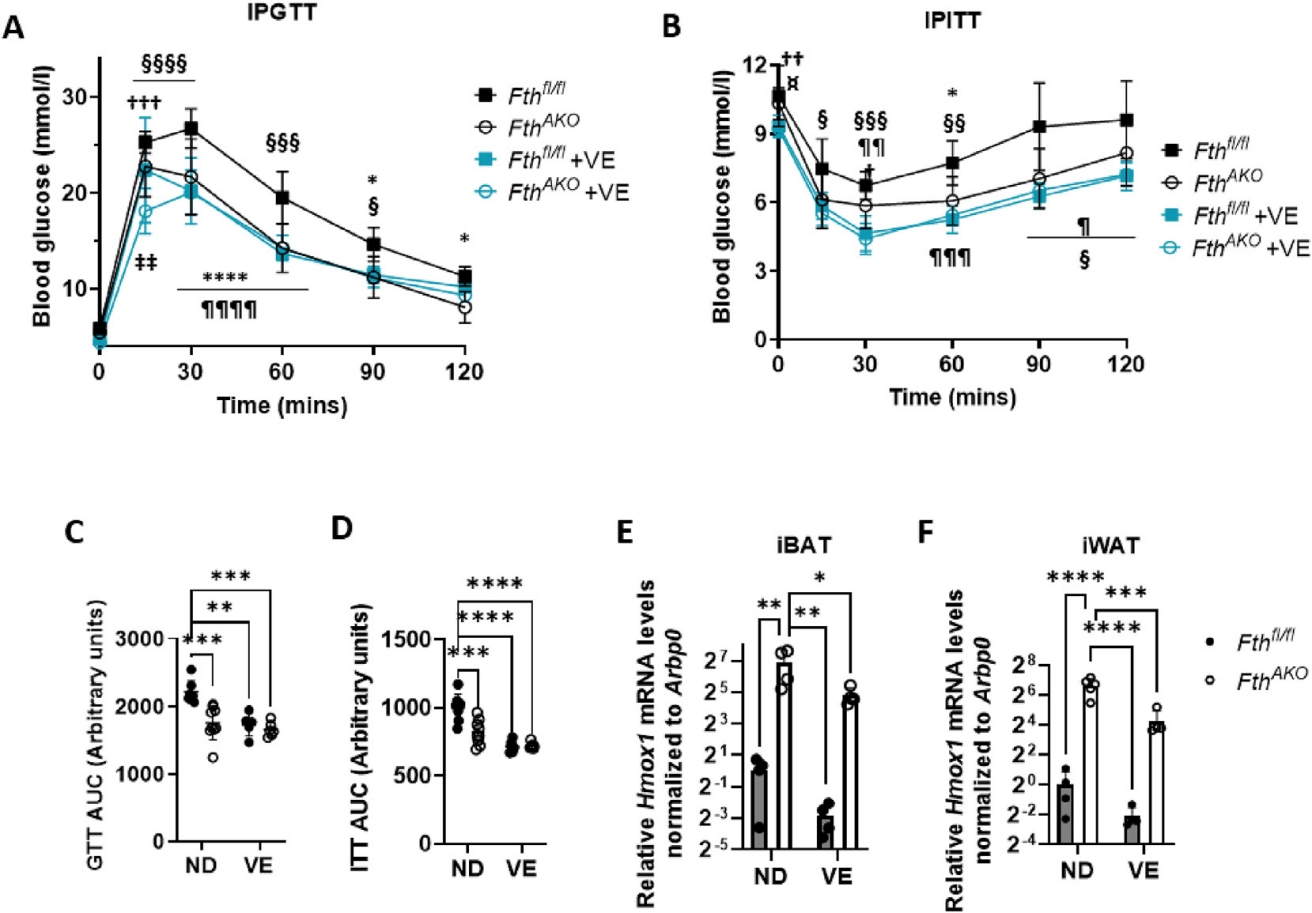
**Vitamin E supplementation improved the glucose tolerance and insulin resistance of both *Fth***^***AKO***^**and control mice and diminished the difference between them**. (A) IPGTT and (B) IPITT results of *Fth*^*AKO*^ and *Fth*^*fl/fl*^ mice supplemented with vitamin E (0.5 g α-tocopherol/kg of food, fed for 3 weeks, *n* = 5–6, from two independent experiments). The IPGTT and IPITT results of normal diet (ND) fed mice were also shown in the figures for comparison (*n* = 7–9). ∗p < 0.05, ∗∗∗∗p < 0.0001 (ND *Fth*^*AKO*^ vs ND *Fth*^*fl/fl*^); †p < 0.05, ††p < 0.01, †††p < 0.001 (ND *Fth*^*AKO*^ vs VE *Fth*^*AKO*^); §p < 0.05, §§p < 0.01; §§§p < 0.001; §§§§p < 0.0001 (ND *Fth*^*fl/fl*^ vs VE *Fth*^*AKO*^); ¶p < 0.05, ¶¶p < 0.01; ¶¶¶p < 0.001, ¶¶¶¶p < 0.0001 (ND *Fth*^*fl/fl*^ vs VE *Fth*^*fl/fl*^); ‡‡p < 0.01 (VE *Fth*^*AKO*^ vs VE *Fth*^*fl/fl*^); ¤ p < 0.05 (ND *Fth*^*AKO*^ vs VE *Fth*^*fl/fl*^). (C,D) AUC calculated from Figure 7A,B. (E,F) qRT-PCR analysis of *Hmox1* expression in iBAT and iWAT of *Fth*^*AKO*^ and *Fth*^*fl/fl*^ mice fed with ND or VE (*n* = 4–5, from three independent experiments). Data from Figure 7A,B was analyzed by two-way ANOVA followed by Sidak's multiple comparisons. Other data was analyzed by two-tailed unpaired t-tests. ∗*p* < 0.05, ∗∗*p* < 0.01, ∗∗∗*p* < 0.001, and ∗∗∗∗*p* < 0.0001.

To verify whether the increment of glucose tolerance and insulin sensitivity in *Fth*^*AKO*^ mice was related to oxidative stress, we fed both *Fth*^*AKO*^ and control mice with a diet supplemented with an antioxidant, vitamin E (0.5 g α-tocopherol/kg of food), for three weeks and performed IPGTTs and IPITTs on them. Glucose tolerance and insulin sensitivity were dramatically increased in vitamin E-supplemented mice, both in *Fth*^*AKO*^ and control mice, compared to *Fth*^*fl/fl*^ fed with a normal diet (ND) (Figure 7A–D). More importantly, the difference in glucose tolerance and insulin sensitivity between *Fth*^*AKO*^ and control mice was eliminated by vitamin E treatment. To confirm that excess vitamin E indeed reduced oxidative stress in AT, we examined the expression level of *Hmox1*. In iBAT and iWAT of vitamin E-supplemented mice, the expression of *Hmox1* was significantly reduced (Figure 7E,F). Previous studies suggest that ROS can both enhance and reduce insulin sensitivity [43,44]. Our results indicate that vitamin E supplementation reduced oxidative stress and promoted insulin sensitivity in both *Fth*^*AKO*^ and control mice. Moreover, vitamin E attenuated the difference in insulin sensitivity between ND-fed *Fth*^*AKO*^ and control mice, suggesting that the original difference is connected to oxidative stress.

## 4 Discussion

The role of ferritin in influencing glucose and lipid metabolism in adipose tissue is not known. Here we report that ferritin in adipocytes plays an important role in maintaining adipocyte iron homeostasis and whole-body glucose metabolism. Loss of Fth in adipocytes reduced intracellular iron storage and slightly increased the labile iron level and oxidative stress, but dramatically induced the expression of *Hmox1*, concomitant with enhanced mitochondrial ETC gene expression and respirational capability. *Fth*^*AKO*^ mice exhibited increased systemic glucose tolerance and insulin sensitivity, shifting substrate preference toward fats from carbohydrates. While some aspects of metabolic disruption were pronounced, we noticed others only partially changed. We attribute this to the mild shift of iron and ROS metabolism due to FTH loss in the adipocyte. It is possible that some genes, such as *Hmox1,* are more sensitive to the change than others.

Ferritin knockout resulted in two major alterations. One is mildly elevated cytosolic iron and ROS, and another is strengthened mitochondrial functions. The latter might be the result of elevated labile iron because more iron available may enhance the formation of ETC complexes and, consequently, activity. Either one or a combination of both changes may be responsible for the observed improved energy metabolism phenotype. Increased ROS has been reported to be beneficial for adipose physiology [45]. On the other hand, more robust mitochondria favor fatty acid oxidation or oxygen consumption, allowing more glucose conversion to fatty acids.

Elevated cytosolic iron led to increased ROS in the cytosol. However, in the mitochondria, ROS was reduced. Studies report that they have opposite effects on mitochondrial function and animal lifespans [46,47]. What has caused the decreased mtROS? We speculate that the increased mitochondrial respiring capability, enabled by increased Fe–S cluster synthesis and enhanced ETC complex's activity, might have dampened the mtROS generation. Alternatively, it has been reported that cytosolic ROS-induced HO-1 could enter mitochondria and reduce mtROS [48].

In agreement with other *Fth* knockout or knockdown models, deletion of *Fth* in adipocytes reduces intracellular iron storage, increases cytosolic LIP, and oxidative stress via changes in the expression of some iron-responsive genes or activity [23,24,49,50], suggesting the iron storage function is closely tied with the protective function against oxidative stress. FTH ablation in this study does not cause severe iron deficiency or iron overload in ATs, which is likely due to the compensatory functions of other iron homeostasis genes as well as local iron exchange, facilitated by iron and ferritin exchange of adipocytes with local macrophage [27,51]. Meanwhile, iron distribution in cellular compartments is perturbed in FTH-deficient AT. The increased aconitase activity suggests increased Fe–S cluster assembly, implying sufficient iron supply, which is in line with the results of LIP measurements in iBAT. Consistently, activity assays of mitochondrial ETC complex I and II and NADH consumption assay suggest augmented mitochondrial ETC function and more active energy metabolism [52,53].

Our data shows that *Fth* deletion leads to statistically valid changes in cytosolic ROS, LIP, and aconitase activity only in iBAT and not in iWAT. A plausible explanation of such differential effects of the *Fth* gene knockout is the large difference in iron demand and iron flux between brown fat and white fat [54,55]. Due to the energy metabolism and thermogenic function of iBAT, a large amount of iron is required; on the contrary, high iron flux is not essential in iWAT.

It is well established that iron in adipocyte tissues plays a crucial role in supporting mitochondrial biogenesis and regulating organismal thermogenesis [3,11,56]. Studies have shown that iron is necessary for adipocyte differentiation and the thermogenic function [11,56,57]. Iron deficiency can impair the formation of beige adipocytes and reduce the thermogenic ability of brown adipocytes [2,4]. Adipocyte-specific deletion of transferrin receptor 1 (*Tfrc*) in adipocytes shows reduced iron content in AT, damaged mitochondrial function, and impaired brown fat thermogenesis [4,9]. In this study, *Fth* deletion in AT increases the expression of some mitochondrial ETC genes and white fat browning marker genes, leading to enhanced heat-generating capacity and intensified adaptive thermogenesis. Adaptive thermogenesis is also improved in *Fth*^*AKO*^ mice, as indicated by the higher body temperature under cold stress. However, the gene expression of *Ucp1* is not induced compared to controls. We explain that *Ucp1* expression does not directly lead to the increase in thermogenesis; Instead, it might be more activated in *Fth1* knockout BAT [58]. Further investigation is needed to elucidate the mechanism of increased adaptive thermogenesis in *Fth*^*AKO*^ mice.

Many previous *Fth* gene knockout studies have reported the phenotype of upregulated *Hmox1* expression and attributed this to oxidative stress [22,59,60]. *Hmox1* responds to various stimuli, including but not limited to ROS, hypoxia, heme, free fatty acids, and heavy metals. Our results suggest that elevated *Hmox1* expression under FTH deficiency is also induced by ROS rather than hypoxia or heme, though we could not rule out other possibilities. The observation that no appreciable lipid peroxidation damage is detected could be attributed to the fact that the very mild oxidative stress might be well protected by oxidative response genes such as *Hmox1* induction to prevent and mitigate lipid oxidation damage. In other words, the upregulation of *Hmox1* expression may compensate for the antioxidant function of ferritin. It is also possible that upregulation of *Hmox1* expression occurs in AT macrophages, and accelerated heme recycling helps reduce ROS and damage in the adipocytes [61]. The upregulated *Hmox1* has been proven to improve mitochondrial biogenesis and ETC gene expression [[32], [33], [34]], consistent with our findings.

Our data suggests that the expression of several adipokines is upregulated in ATs of *Fth*^*AKO*^ mice. The upregulated expression of *AdipoQ* may be related to increased mitochondrial function and *Hmox1* [62]. Increased mitochondrial biogenesis and function may be related to adiponectin synthesis [63]. Despite one research that disagrees with a direct HO-1-adiponectin axis hypothesis [64], massive studies provide evidence of simultaneous upregulation of the two genes [[65], [66], [67]]. In FTH-deleted iWAT, the qRT-PCR analyses demonstrate the activation of both browning and lipogenesis. Browning of WAT is often related to increased lipolysis and energy expenditure, which seems to conflict with lipogenesis. However, several studies report simultaneous up- [68,69] or down-regulated [70] browning and lipogenesis. The increased insulin sensitivity is aligned with upregulated lipogenesis genes and *Lep* in the iWAT of the *Fth*^*AKO*^ mice. Meanwhile, the body weights and fat sizes show no difference between the *Fth*^*AKO*^ and control mice, which demonstrates the net lipid gain is comparable. We speculate that lipogenesis, browning, and lipid turnover are enhanced and reach a new steady state in FTH-deleted iWAT. The observation of synchronously upregulated *Fabp4* and *Lep* expression suggested a sign of hypoxia [71,72] or inflammation [73,74], but we did not see any increase in hypoxia or inflammation markers in our study. Further studies are needed to clarify the mechanisms.

It has become a consensus that ROS causes insulin resistance. However, a growing number of studies suggest that mildly increased ROS could improve insulin sensitivity [44,75]. In this study, we found no lipid peroxidation damage in Fth-deficient AT, and the cytosolic ROS increment is moderate. An appropriate increase in ROS is also beneficial for the thermogenic functions of mitochondria. Coupled with the protective effect of induced antioxidant genes in FTH-deleted AT, we believe that the slightly increased ROS may have beneficial effects on insulin sensitivity and thermogenesis. Vitamin E is a potent antioxidant and ROS scavenger. Up to now there is no consistent conclusion on the effect and mechanism of vitamin E on mice's insulin sensitivity [[76], [77], [78], [79]]. Our data suggests that vitamin E robustly reduces oxidative stress in ATs and promotes insulin sensitivity in both *Fth*^*AKO*^ and control mice. Additionally, vitamin E supplementation erases the difference in insulin sensitivity between *Fth*^*AKO*^ and control mice, further supporting that the enhanced insulin sensitivity caused by *Fth*^*AKO*^ is associated with oxidative stress. Both slight ROS elevation and ROS mitigation may promote insulin sensitivity: in *Fth*^*AKO*^, a slight ROS increase may underlie the observed insulin sensitivity and glucose tolerance, but in vitamin E-treated mice, ROS is both suppressed in *Fth*^*AKO*^ and control mice, leading to a further increment of insulin sensitivity and glucose tolerance in them. Noteworthy is that *Hmox1* expression is not completely normalized after vitamin E treatment. This raises the question of how *Hmox1* was induced in *Fth*^*AKO*^, whether vitamin E completely suppressed the ROS or even more, and whether vitamin E had other mechanisms regulating insulin sensitivity in addition to neutralizing the ROS.

Studies have shown a cross-talk between iron and glucose metabolism [1]. Iron may indirectly interfere with glucose metabolism and insulin resistance [80,81]. Meanwhile, iron level affects the synthesis and the release of adipokines, including adiponectin and leptin, which have a strong influence on glucose metabolism and insulin resistance [17,82,83]. As we observed a manifest increase in glucose tolerance and insulin sensitivity in addition to changes in the expression of several adipokines as the consequence of adipocyte-specific *Fth1* knockout, we believe overall the level of free iron is well regulated and the beneficial consequences caused by *Fth1* deletion, including mild ROS increment, *Hmox1* induction, and mitochondrial function improvement, overrides possible detrimental consequences caused by slightly increased LIP.

In conclusion, our study provides valuable insights into the role of FTH in adipocytes in modulating glucose and lipid metabolism via influencing iron homeostasis and mitochondrial function. By eliminating *Fth* expression, we observed significant alterations in glucose tolerance, insulin sensitivity, and adaptive thermogenesis. Our findings suggest that ferritin may serve as a potential therapeutic target for obesity and diabetes. However, the mechanism of ferritin or iron regulating some of the adipokines remains unclear and warrants further investigation.

## CRediT authorship contribution statement

**Binyu Lu:** Data curation, Formal analysis, Investigation, Methodology, Validation, Visualization, Writing – original draft. **Shanshan Guo:** Methodology, Resources, Visualization. **Jialin Zhao:** Data curation, Methodology. **Xiaoting Wang:** Methodology. **Bing Zhou:** Conceptualization, Formal analysis, Funding acquisition, Project administration, Supervision, Writing – review & editing.

## Declaration of competing interest

The authors declare that they have no known competing financial interests or personal relationships that could have appeared to influence the work reported in this paper.

## Data Availability

Data will be made available on request.
