# Supplementary material for: Adipose knockout of H-ferritin improves energy metabolism in mice
Source: Mol Metab. 2024 Jan 5;80:101871. doi: 10.1016/j.molmet.2024.101871 (PMC10803945; doi:10.1016/j.molmet.2024.101871)
Supplement: Multimedia component 1 [file mmc1.docx]

**Appendix A. Supplementary data**


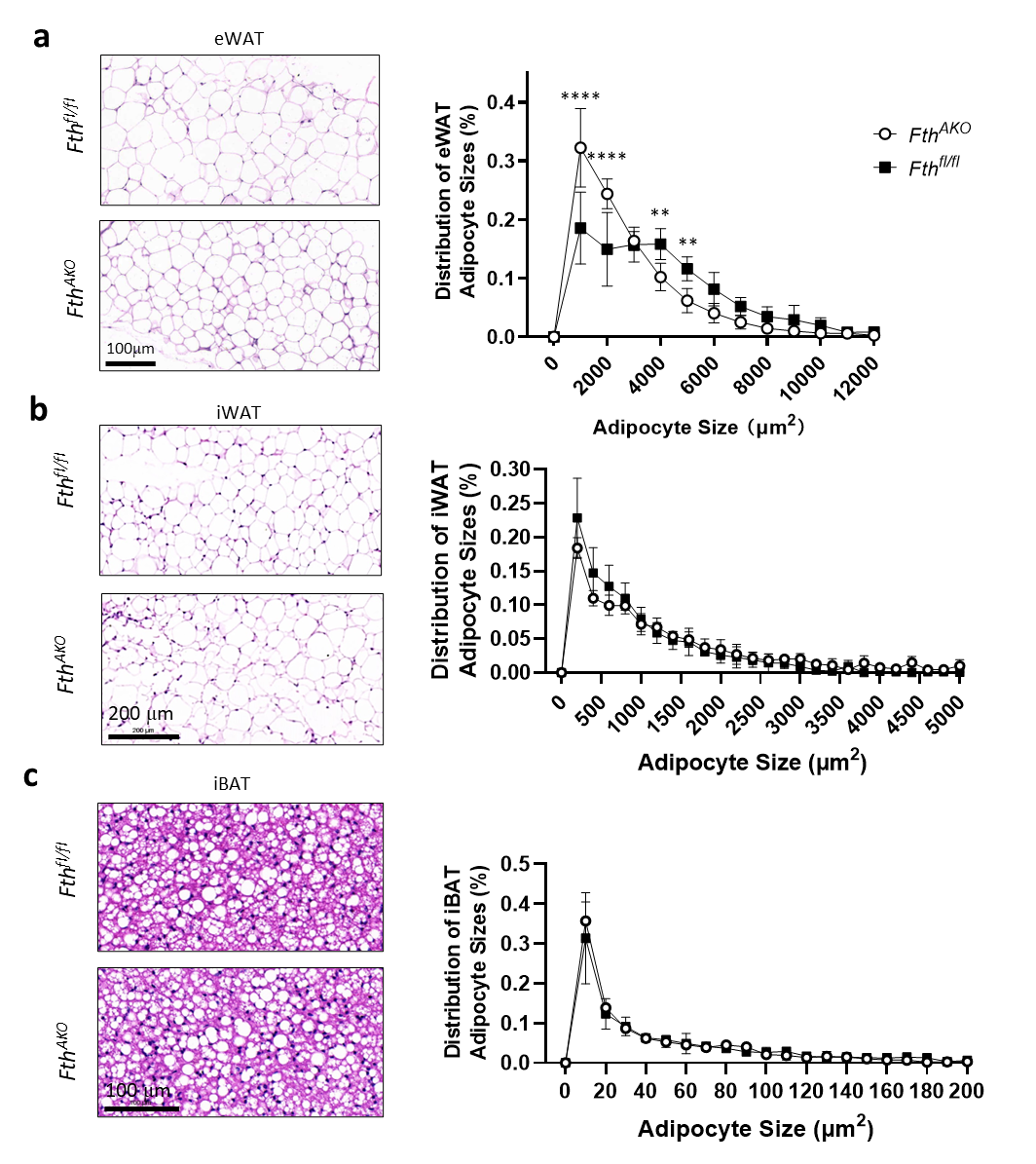


**Fig.S1 Adipocyte size was reduced in eWAT of *Fth^AKO^* mice.** The representative images and the quantification results of adipocyte sizes of H&E staining of eWAT (A), iWAT (B), and iBAT (C) (*n* = 3-4 mice, 5 random images for each mouse were taken for quantification). Adipocyte sizes were analyzed by multiple two-tailed unpaired t-tests. ***p* < 0.01 and *****p* < 0.0001.


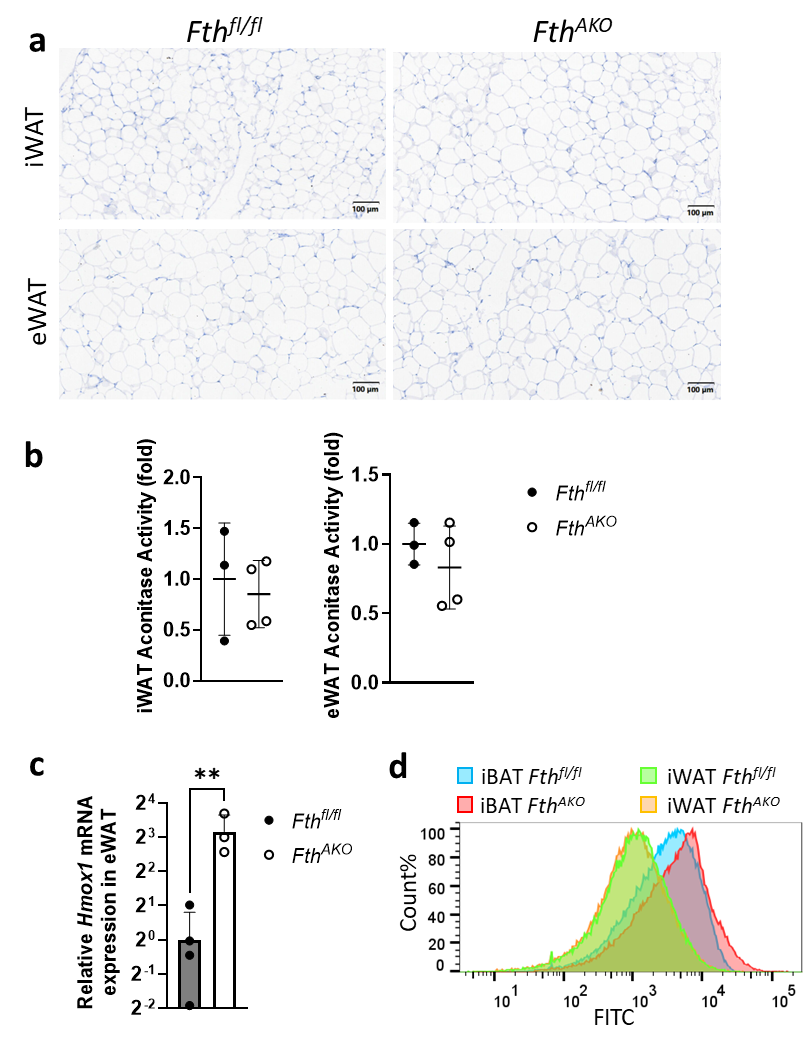


**Fig.S2** **Alternations of iron homeostasis and oxidative stress in the ATs of *Fth^AKO^* mice.** (A) Representative images of Perl’s and DAB iron staining (brown) and Hematoxylin (blue) staining of ATs. (B) Results of aconitase activity assay of iWAT and eWAT (*n* = 3-4, from two independent experiments). (C) *Hmox1* mRNA expression measured by qRT-PCR assay, normalized to the expression of *Arbp0* (*n* = 3-4). (D) A representative flow cytometry result of cytosolic ROS measurement detected by the DCFH-DA probe. Data was analyzed by two-tailed unpaired t-tests. ***p* < 0.01

**Table S1. qRT-PCR primer sequences.**

|  |  |  |
| --- | --- | --- |
|  | forward | reverse |
| *18S* | TTCTGGCCAACGGTCTAGACAAC | CCAGTGGTCTTGGTGTGCTGA |
| *AdipoQ* | AGATGCAGGTCTTCTTGGTC | TCTCCAGGCTCTCCTTTCC |
| *Arbp0* | CTTTGGGCATCACCACGAA | GCTGGCTCCCACCTTGTCT |
| *Atgl* | TTCGCAATCTCTACCGCCTC | AAAGGGTTGGGTTGGTTCAG |
| *Atp5a1* | TCTCCATGCCTCTAACACTCG | CCAGGTCAACAGACGTGTCAG |
| *C/ebp-α* | TGGACAAGAACAGCAACGAG | TCACTGGTCAACTCCAGCAC |
| *Car4* | CAGAGCACAGTATTGATGGGAG | CTTGTTCACCTTGTCTCCTACC |
| *CD137* | AGGAGCTAACGAAGCAGGGTTG | TCCCGGTCTTAAGCACAGACCTTC |
| *Cidea* | ATCACAACTGGCCTGGTTACG | TACTACCCGGTGTCCATTTCT |
| *Cox4* | ATGTCACGATGCTGTCTGCC | GTGCCCCTGTTCATCTCGGC |
| *Cox8b* | GAACCATGAAGCCAACGACT | GCGAAGTTCACAGTGGTTCC |
| *CPT1a* | TCTTGCAGTCGACTCACCTT | TCCACAGGACACATAGTCAGG |
| *CPT1b* | ATGTATCGCCGCAAACTGGACC | CTCTGAGAGGTGCTGTAGCAAG |
| *Dio2* | CTTCCTCCTAGATGCCTACAAAC | TCTCCGAGGCATAATTGTTACC |
| *Elovl3* | TACATCTGGAGGCAGGAGAA | GGTGGAAGAAGTGAGCGAATAG |
| *Fabp4* | ACACCGAGATTTCCTTCAAACTG | CCATCTAGGGTTATGATGCTCTTCA |
| *Fpn* | TTGCAGGAGTCATTGCTGCTA | TGGAGTTCTGCACACCATTGAT |
| *Fth* | GAGCCCTTTGCAACTTCGTC | TCATCACGGTCTGGTTTCTTT |
| *Glut4* | GTGACTGGAACACTGGTCCTA | CCAGCCACGTTCATTGTAG |
| *Gpx4* | CCTCCCCAGTACTGCAACAG | GGCTGAGAATTCGTGCATGG |
| *Hif1α* | CAGAATGCTCAGAGGAAGCG | CTGCATGCTAAATCGGAGGG |
| *Hmox1* | TGACACCTGAGGTCAAGCAC | TCTCTGCAGGGGCAGTATCT |
| *Hsl* | GCTGGGCTGTCAAGCACTGT | GTAACTGGGTAGGCTGCCAT |
| *Il-6* | AGAGACTTCCATCCAGTTGCCT | TCTGTTGGGAGTGGTATCCTCTGT |
| *Lep* | TGAAGCCCAGGAATGAAGTC | TCAAGACCATTGTCACCAGG |
| *Mgl* | AGGCGAACTCCACAGAATGTT | ACAAAAGAGGTACTGTCCGTCT |
| *Ndufs1* | AGGATATGTTCGCACAACTGG | TCATGGTAACAGAATCGAGGGA |
| *Nqo1* | CAGATCCTGGAAGGATGGAA | TCTGGTTGTCAGCTGGAATG |
| *Nrf1* | CCAGVAAGTCCAGCAGGTCC | TTCCCTGTTGCCACAGCAGC |
| *Nrf2* | TCTTGGAGTAAGTCGAGAAGTGT | GTTGAAACTGAGCGAAAAAGGC |
| *Pgc1-α* | AGCCGTGACCACTGACAACGAG | GCTGCATGGTTCTGAGTGCTAAG |
| *Plin1* | CTGTGTGCAATGCCTATGAGA | CTGGAGGGTATTGAAGAGCCG |
| *Ppar-γ* | GTGCCAGTTTCGATCCGTAGA | GGCCAGCATCGTGTAGATGA |
| *Prdx1* | AGTCCAGGCCTTCCAGTTCACT | GGCTTGATGGTATCACTGCCAG |
| *Retn* | AAGAACCTTTCATTTCCCCTCCT | GTCCAGCAATTTAAGCCAATGTT |
| *Scd1* | TTCTTGCGATACACTCTGGTGC | CGGGATTGAATGTTCTTGTCGT |
| *Sdhc* | GCTGCGTTCTTGCTGAGACA | ATCTCCTCCTTAGCTGTGGTT |
| *Serca2* | TCGACAGGACAGAAAGAGTGTG | AAACTGAATTCAACTCACCAGC |
| *Sod1* | GAGCATTCCATCATTGGCCG | TTCCACCTTTGCCCAAGTCA |
| *Sod2* | CCCAAAGGAGAGTTGCTGGAGG | GCTCCCACACGTCAATCCCC |
| *Tfrc1* | GTTTTTGTGAGGATGCAGACTATCC | GCTGAGGAACTTTCTGAGTCAATG |
| *Tnf-α* | AGGGTCTGGGCCATAGAACT | CCACCACGCTCTTCTGTCTAC |
| *Ucp1* | CACCTTCCCGCTGGACACT | CCCTAGGACACCTTTATACCTAATGG |
